# Mechanisms underlying the reversal of hypoxia-induced energy metabolic reprogramming in cardiomyocytes by Poria cocos triterpenoids: integrated regulation of mitochondrial dynamics and the autophagy–lysosomal pathway

**DOI:** 10.3389/fcvm.2026.1891480

**Published:** 2026-08-27

**Authors:** Zuoyue Wu, Jiadan Liao, Huilei Xu, Hongwei Hou, Jia Song

**Affiliations:** 1 Department of Cardiology, The Third Affiliated Hospital of Zhejiang Chinese Medical University, Hangzhou, China; 2 Department of Ophthalmology, Zhejiang Greentown Cardiovascular Hospital, Hangzhou, China

**Keywords:** autophagy-lysosomal pathway, energy metabolic reprogramming, hypoxia, mitochondrial dynamics, Poria triterpenoids

## Abstract

**Objective:**

To elucidate the mechanisms by which Poria cocos triterpenoids reverse hypoxia-induced energy metabolic reprogramming in cardiomyocytes through integrated regulation of mitochondrial dynamics and the autophagy–lysosomal pathway.

**Methods:**

A hypoxic injury model was established in H9c2 rat cardiomyoblasts. Cells were randomized into five groups: normal control, hypoxia model, and low-, medium-, and high-dose Poria cocos triterpenoid–treated groups. Cell viability was assessed using the Cell Counting Kit-8 (CCK-8) assay. Western blotting quantified protein expression levels of mitochondrial fusion (Mfn1, Mfn2, OPA1) and fission (Drp1, Fis1) regulators; autophagy–lysosomal markers (LC3-II/I ratio, p62, PINK1, Parkin, LAMP1, ATG5); and key metabolic enzymes (CPT1, PFK-2). Mitochondrial respiratory control ratio (RCR) and ATP production rate were measured via high-resolution respirometry; extracellular acidification rate (ECAR), oxygen consumption rate (OCR), and fatty acid oxidation (FAO) efficiency were determined using Seahorse XF Analyzer. Intracellular lactate and pyruvate concentrations were quantified enzymatically. Functional validation employed siRNA-mediated knockdown of Drp1 or ATG5 to assess mechanistic dependency.

**Results:**

Compared with the normal control group, the hypoxia model group exhibited significantly reduced cell viability (*P* < 0.05), impaired mitochondrial fusion (downregulated Mfn1, Mfn2, OPA1) and enhanced fission (*P* < 0.05). Mitochondrial bioenergetics were compromised: RCR, ATP production rate, OCR, FAO efficiency, CPT1 activity, and pyruvate levels decreased (*P* < 0.05), whereas lactate accumulation, ECAR, and PFK-2 activity increased (*P* < 0.05), indicating a glycolytic shift. Autophagy–lysosomal flux was suppressed: LC3-II/I ratio, PINK1, Parkin, and LAMP1 expression declined, while p62 accumulated (*P* < 0.05). Treatment with Poria cocos triterpenoids dose-dependently reversed these alterations: viability improved; fusion proteins increased and fission proteins decreased (*P* < 0.05); mitochondrial respiration, ATP synthesis, FAO efficiency, and pyruvate levels recovered; glycolytic parameters normalized; and autophagic flux was restored (elevated LC3-II/I, PINK1, Parkin, LAMP1; reduced p62, with maximal effects observed at the high dose (*P* < 0.05). Mechanistic validation confirmed that Drp1 knockdown synergistically enhanced the protective effects of triterpenoids—further improving OCR, ATP production, FAO efficiency, and CPT1 activity while suppressing ECAR, lactate, and PFK-2 activity (*P* < 0.05). In contrast, ATG5 knockdown abolished triterpenoid-mediated rescue of viability, bioenergetics, and metabolic phenotype (*P* < 0.05), confirming ATG5-dependent autophagy as essential for the therapeutic effect.

**Conclusion:**

Poria cocos triterpenoids restore energy homeostasis in hypoxic cardiomyocytes by concurrently normalizing Drp1-mediated mitochondrial fission and ATG5-dependent autophagic clearance. This dual regulation rescues mitochondrial structural integrity, enhances oxidative phosphorylation, suppresses pathological glycolysis, and thereby reverses hypoxia-induced metabolic reprogramming—ultimately preserving cardiomyocyte viability.

## 1 Introduction

Cardiovascular diseases remain a leading global cause of mortality and morbidity. Mitochondrial dysfunction is a well-established pathogenic driver in myocardial ischemia, heart failure, and metabolically induced cardiomyopathy (1, 2). Cardiomyocytes exhibit exceptionally high energy demands and rely predominantly on mitochondrial oxidative phosphorylation (OXPHOS) for ATP generation. Beyond bioenergetics, mitochondria critically regulate redox homeostasis, calcium buffering, and cell fate decisions—including apoptosis and ferroptosis (3). Hypoxia disrupts mitochondrial OXPHOS efficiency, elevates reactive oxygen species (ROS) production, and triggers a profound shift in substrate utilization—from fatty acid oxidation (FAO) toward accelerated glycolysis. These perturbations collectively impair cellular integrity and contractile function (4).

Mitochondrial homeostasis is dynamically maintained through two interdependent processes: mitochondrial dynamics (fission and fusion) and the autophagy–lysosomal pathway—particularly mitophagy, the selective autophagic clearance of damaged mitochondria (5). Fusion proteins—including mitofusins 1 and 2 (Mfn1, Mfn2) and optic atrophy 1 (OPA1)—preserve mitochondrial network integrity and respiratory competence, whereas the fission GTPase Drp1, recruited by adaptors such as Fis1, mediates mitochondrial division to facilitate quality control (6). Hypoxic stress disrupts this equilibrium, promoting excessive Drp1-dependent fission, mitochondrial fragmentation, loss of membrane potential (Δ*Ψ*m), and respiratory deficiency (7). Mitophagy acts as a critical surveillance mechanism: PINK1 accumulation on depolarized mitochondria recruits Parkin to ubiquitinate outer membrane proteins, enabling LC3-mediated engulfment and lysosomal degradation via LAMP1-positive vesicles (8). Impairment of autophagic flux or lysosomal degradation leads to accumulation of dysfunctional mitochondria and metabolic debris, thereby amplifying ROS, exacerbating energetic crisis, and accelerating cardiomyocyte injury (9).

Concomitant with structural and functional deterioration, hypoxia induces a hallmark metabolic reprogramming in cardiomyocytes: under normoxia, >70% of ATP derives from FAO and OXPHOS; during sustained hypoxia, cells undergo a “glycolytic switch”, characterized by reduced oxygen consumption rate (OCR), elevated extracellular acidification rate (ECAR), lactate accumulation, and diminished ATP yield per glucose molecule (10). Critically, this metabolic shift is not isolated—it is mechanistically coupled to dysregulated mitochondrial dynamics and defective mitophagy, forming a self-perpetuating cycle that sustains mitochondrial dysfunction and compromises energy supply (11).

Poria cocos (syn. Wolfiporia extensa), a widely used traditional Chinese medicinal fungus, contains polysaccharides, sterols, and triterpenoids as its principal bioactive constituents—with triterpenoids (e.g., poricoic acids, dehydrotumulosic acid, and pachymic acid) representing key pharmacologically active molecules (12, 13). Modern pharmacological studies demonstrate that Poria cocos triterpenoids exert anti-inflammatory, antioxidant, glucose- and lipid-metabolism–modulating, and multi-organ protective effects (14). Notably, poricoic acid A has been shown to attenuate hypoxia-induced cardiomyocyte injury by activating the AMPK/PGC-1*α*/SIRT3 axis, thereby restoring mitochondrial biogenesis, reducing oxidative stress, and improving respiratory capacity (15–17). These findings strongly suggest that Poria cocos triterpenoids confer cardioprotection—at least in part—by targeting mitochondrial homeostasis. However, whether these compounds reverse hypoxia-induced *energy metabolic reprogramming* through *coordinated regulation of mitochondrial dynamics and the autophagy–lysosomal pathway* remains unexplored.

To address this gap, we employed an established H9c2 rat cardiomyoblast hypoxia model and systematically investigated: (i) alterations in mitochondrial fission/fusion balance (Drp1, Fis1, Mfn1, Mfn2, OPA1); (ii) autophagic flux and lysosomal functionality (LC3-II/I ratio, p62, PINK1, Parkin, LAMP1, ATG5); and (iii) comprehensive bioenergetic profiling (OCR, ECAR, FAO efficiency, RCR, ATP production rate, lactate/pyruvate ratio, CPT1 and PFK-2 activities). Our findings provide mechanistic evidence that Poria cocos triterpenoids rescue hypoxic cardiomyocytes by concurrently normalizing Drp1-mediated fission and ATG5-dependent autophagic clearance—thereby breaking the vicious cycle of mitochondrial damage, metabolic inflexibility, and energetic failure (18).

### 1.1 Experimental materials

#### 1.1.1 Cell culture

Rat cardiomyoblasts (H9c2, ATCC® CRL-1446™) were cultured in high-glucose Dulbecco's Modified Eagle Medium (DMEM; Gibco, USA) supplemented with 10% (v/v) heat-inactivated fetal bovine serum (FBS; Gibco, Cat#10099141) and 1% (v/v) penicillin–streptomycin solution (Gibco, Cat#15140122) at 37 °C in a humidified atmosphere of 5% CO₂ (Heracell VIOS 160i, Thermo Fisher Scientific, USA). Cells were passaged using 0.25% trypsin–EDTA (Gibco, Cat#25200056) upon reaching 80–90% confluence. All experiments utilized cells in the logarithmic growth phase (passages 4–8) to ensure phenotypic consistency and metabolic responsiveness.

#### 1.1.2 Preparation and treatment of Poria cocos triterpenoids

A standardized triterpenoid extract from Poria cocos (total triterpenoid content ≥98%, Chengdu Mansite Biotechnology Co., Ltd., China) was dissolved in dimethyl sulfoxide (DMSO; Sigma-Aldrich, Cat#D2650) to prepare a 100 mg/mL stock solution. Working dilutions (25, 50, and 100 μg/mL) were prepared in serum-free DMEM immediately before use. Final DMSO concentration was maintained at ≤0.1% (v/v) across all groups—including controls—to exclude solvent-related artifacts. Hypoxia-mimetic injury was induced using cobalt(II) chloride hexahydrate (CoCl₂·6H₂O; Sigma-Aldrich, Cat#C8661) at 600 μM for 24 h—a concentration and duration validated to reproducibly suppress HIF-prolyl hydroxylase activity, stabilize HIF-1*α*, and recapitulate key features of hypoxic metabolic reprogramming without inducing acute cytotoxicity.

The triterpenoids in this study are derived from Poria cocos triterpene extract, with a molecular formula of C₃₃H₅₂O₅ and a molecular weight of 528.76. Physicochemically, these compounds generally exhibit no significant ultraviolet absorption, showing only terminal absorption near 200 nm. They produce characteristic color reactions when reacted with strong acids or Lewis acids under anhydrous conditions, and methanol provides the best solubility. Existing studies confirm that the optimal extraction method for these compounds is using 85% ethanol at 55°C for one hour. For storage, they should be kept sealed, protected from light, and stored at 0–10 °C.

#### 1.1.3 Experimental design and grouping

This study employed a sample size estimation logic based on a multi-group equal design. The preset significance level was *α* = 0.05, and the statistical power was 1 - *β* = 0.8. Combined with the difference in the effect of polyphyllin from Poria cocos in improving CoCl₂-induced cell damage in the pre-experiment, the chi-square distribution non-central parameter *λ* threshold value table was consulted for correction. At the same time, considering the parallel sample loss during cell culture operations and the within-group dispersion, it was finally determined that each group should have 6 parallel culture dishes, which not only met the statistical test power but also was suitable for the operational feasibility of the 5-group experiment. Cells were randomized into five experimental groups (*n* = 6 per group per assay): (i) Normal control: cultured in complete medium + 0.1% DMSO for 24 h; (ii) Hypoxia model: cultured in complete medium + 600 μM CoCl₂ + 0.1% DMSO for 24 h; (iii) Low-dose triterpenoid: complete medium + 600 μM CoCl₂ + 25 μg/mL triterpenoids; (iv) Medium-dose triterpenoid: complete medium + 600 μM CoCl₂ + 50 μg/mL triterpenoids; (v) High-dose triterpenoid: complete medium + 600 μM CoCl₂ + 100 μg/mL triterpenoids. All treatments were performed for 24 h under standard culture conditions (37°C, 5% CO₂).

### 1.2 Methods

#### 1.2.1 Cell viability assessment (CCK-8 assay)

H9c2 cells were seeded in 96-well plates (Corning, USA) at 5 × 10^3^ cells/well (five technical replicates/group). Following treatment, 10 μL of CCK-8 reagent (Dojindo, Cat#CK04) was added per well, and plates were incubated for 2 h at 37 °C. 2103;. Absorbance at 450 nm was measured using a SpectraMax i3x multimode microplate reader (Molecular Devices, USA). Cell viability (%) was calculated as: (OD₄₅₀,treatment/OD₄₅₀, normal control) × 100. Data represent the mean ± SD of three independent biological replicates.

#### 1.2.2 Western blot analysis

After treatment, cells were washed twice with ice-cold PBS and lysed on ice for 30 min in RIPA buffer (Beyotime, China) containing 1 mM PMSF and protease/phosphatase inhibitor cocktails (Roche, Switzerland). Lysates were centrifuged at 12,000 × g, 4℃ for 15 min; supernatants collected and protein concentration determined by BCA assay (Thermo Fisher Scientific, USA). Equal amounts (40 μg) of total protein were resolved by SDS-PAGE and transferred onto PVDF membranes (Millipore, USA). Membranes were blocked with 5% non-fat milk in TBST for 1 h at room temperature, then probed overnight at 4 ℃ with primary antibodies against: Mfn1, Mfn2, OPA1, Drp1, Fis1 (mitochondrial dynamics); LC3-II/I, p62, PINK1, Parkin, LAMP1, ATG5 (autophagy–lysosomal pathway); and β-actin (loading control) (all antibodies from Cell Signaling Technology, USA, unless otherwise specified). After washing, membranes were incubated with HRP-conjugated secondary antibodies (1:5000, Proteintech, USA) for 1 h at room temperature. Immunoreactive bands were visualized using enhanced chemiluminescence (ECL) reagents (Beyotime, China) and captured with a ChemiDoc MP Imaging System (Bio-Rad, USA). Band intensities were quantified using ImageJ v1.53t; target protein expression was normalized to β-actin and expressed as fold-change relative to the normal control group.

#### 1.2.3 Mitochondrial respiratory control ratio (RCR) and ATP production rate

Mitochondria were isolated from treated cells using the Mitochondria Isolation Kit (Beyotime, Cat#C3601) on ice. Protein concentration of mitochondrial suspensions was determined by BCA assay. For high-resolution respirometry, 100 μg mitochondrial protein was added to the Oxygraph-2k chamber (Oroboros Instruments, Austria) containing MiR05 respiration medium (maintained at 37 ℃). Sequential substrate–inhibitor titrations were performed: glutamate (10 mM) + malate (2 mM) to induce Complex I–linked respiration; ADP (2.5 mM) to stimulate phosphorylating respiration; oligomycin (2 μM) to inhibit ATP synthase and assess proton leak. RCR was calculated as State 3/State 4o. ATP production rate was measured using the ATP Assay Kit (Beyotime, Cat#S0026). Luminescence was recorded on a SpectraMax i3x luminometer following luciferase reaction; ATP concentration was interpolated from a standard curve and normalized to mitochondrial protein content, reported as nmol ATP·min⁻^1^·mg⁻^1^ mitochondrial protein.

#### 1.2.4 Lactate and pyruvate quantification

Cell culture supernatants were collected and centrifuged at 3,000 × g, 4 ℃ for 10 min to remove debris. Lactate and pyruvate concentrations were measured enzymatically using commercial assay kits (Nanjing Jiancheng Bioengineering Institute, Cat#A019-2-1 and Cat#A081-1-1, respectively) according to the manufacturer's protocols. Absorbance was read at 530 nm (lactate) and 505 nm (pyruvate) on a SpectraMax i3x microplate reader. Metabolite levels were normalized to total cellular protein content (determined by BCA assay) and expressed as μmol/mg protein.

#### 1.2.5 Seahorse XF metabolic flux analysis

H9c2 cells were seeded at 1 × 10⁴ cells/well in XF96 microplates (Agilent Technologies, USA) and allowed to adhere overnight. After 24 h treatment, culture medium was replaced with Seahorse XF Base Medium (supplemented with 10 mM glucose, 2 mM glutamine, and 1 mM sodium pyruvate; pH 7.4), and plates were equilibrated for 1 h in a CO₂-free incubator at 37 ℃. Mitochondrial stress test (XF Cell Mito Stress Test Kit, Agilent, Cat#103015-100) assessed basal OCR, ATP-linked respiration, maximal respiration, spare respiratory capacity, and non-mitochondrial respiration via sequential injection of oligomycin (1 μM), FCCP (1.5 μM), and rotenone/antimycin A (0.5 μM). Glycolysis stress test (XF Glycolysis Stress Test Kit, Agilent, Cat#103020-100) measured basal ECAR, glycolytic capacity, and glycolytic reserve after injection of glucose (10 mM), oligomycin (1 μM), and 2-deoxyglucose (50 mM). Fatty acid oxidation (FAO) efficiency was evaluated using palmitate–BSA conjugate (Agilent, Cat#103675-100); etomoxir (40 μM, Sigma-Aldrich, Cat#E1905), a specific CPT1 inhibitor, was injected to determine FAO-dependent OCR. FAO efficiency was calculated as: (OCR after palmitate−OCR after etomoxir)/basal OCR × 100%.

#### 1.2.6 Enzymatic activity assays: CPT1 and PFK-2

Cell pellets were lysed in ice-cold lysis buffer (Solarbio, China, for CPT1; Comin Biotechnology, China, for PFK-2). Supernatants were collected after centrifugation (12,000 × g, 4 ℃, 10 min). CPT1 activity was measured spectrophotometrically (450 nm) using the Carnitine Palmitoyltransferase 1 Activity Assay Kit (Solarbio, Cat#BC3545); PFK-2 activity was determined using the 6-Phosphofructokinase-2 Activity Assay Kit (Comin, Cat#COMIN7012). Enzyme activities were calculated from standard curves and normalized to total protein content, reported as U/mg protein (1 U = 1 μmol substrate converted/min).

#### 1.2.7 siRNA-mediated gene silencing and mechanistic validation

H9c2 cells were seeded in 6-well plates and transfected at 50–60% confluence using Lipofectamine 3000 (Thermo Fisher Scientific, USA) with 50 nM Drp1 siRNA (Santa Cruz Biotechnology, USA, sc-35265), ATG5 siRNA (Santa Cruz, sc-41445), or scrambled negative control siRNA (Santa Cruz, sc-37007). Transfection complexes were incubated for 15 min at room temperature prior to addition. After 6 h, medium was replaced with complete medium, and cells were cultured for 48 h to achieve >70% knockdown efficiency (confirmed by Western blot). Subsequently, cells underwent CoCl₂-induced hypoxia ± triterpenoid treatment for 24 h. Knockdown efficacy and downstream effects were assessed by measuring Drp1/ATG5 expression, LC3-II/I ratio, p62 accumulation, cell viability, OCR, ECAR, ATP production rate, FAO efficiency, CPT1/PFK-2 activities, and lactate levels—thereby establishing causal dependency of triterpenoid effects on Drp1 and ATG5 function.

#### 1.2.8 Transmission electron microscopy evaluation of mitochondrial morphology in each group of cells

Cells from each group at the logarithmic growth phase were collected. The original culture medium was removed, and cells were gently rinsed three times with pre-chilled PBS buffer (4 °C) for 3 minutes per rinse to completely eliminate residual medium and serum components. Subsequently, 2.5% glutaraldehyde fixative was added, and cells were fixed in the dark at 4°C for 2 hours. Cells were then lightly scraped off using a cell scraper, centrifuged at 2000rpm for 5 minutes to collect the pellet, and resuspended in fresh 2.5% glutaraldehyde fixative, stored overnight at 4 °C. On the following day, samples were rinsed three times with PBS, followed by postfixation with 1% osmium tetroxide at room temperature in the dark for 1.5 hours. After fixation, samples underwent stepwise dehydration using graded ethanol solutions (30%, 50%, 70%, 90%, and 100%) for 10 minutes each. Then, propylene oxide was used as a transitional agent before embedding in epoxy resin. Polymerization was carried out gradually at 37 °C, 45 °C, and 60 °C to complete block curing. Using an ultramicrotome, the embedded blocks were cut into 70 nm-thick ultrathin sections, which were transferred onto copper grids. Sections were stained sequentially with uranyl acetate and lead citrate solutions for 10 minutes each. Stained samples were examined under a transmission electron microscope at an accelerating voltage of 80 kV. First, low-magnification views were used to locate intact cellular regions, then magnified progressively to an appropriate level. Systematic observation and documentation were performed on mitochondrial double membrane integrity, cristae arrangement and number, degree of mitochondrial swelling, and vacuolization changes. For each group, 20 well-preserved cells were randomly selected to analyze typical ultrastructural features of mitochondria, enabling comparative analysis of morphological differences among groups.

#### 1.2.9 Statistical analysis

In this study, all the data were processed using the SPSS 26.0 statistical analysis software (IBM Corporation, USA); for the measurement data (cell viability, Mfn1, Mfn2, Opa1, Drp1, Fis1, LC3-II/I, PINK1, Parkin, LAMP1 expression, RCR, ATP synthesis rate, pyruvate level, OCR, fatty acid oxidation efficiency, CPT1 activity, lactate level, ECAR and PFK-2 activity), they were expressed as “mean ± standard deviation” ($\bar{x}$±s), and the homogeneity of variance was tested (Levene test). The comparisons between groups were conducted using one-way analysis of variance or repeated measures analysis of variance, and pairwise comparisons between groups were performed using the LSD-t test. A P value < 0.05 indicated a statistically significant difference.

## 2 Results

### 2.1 CCK-8 assay for cell viability of H9c2 cells in each group

Cell viability was assessed using the CCK-8 assay. Compared with the normal control group, the hypoxia model group exhibited a significant reduction in viability (*P* < 0.05). Treatment with Poria cocos triterpenoids dose-dependently attenuated this hypoxia-induced cytotoxicity: viability was significantly higher in the low-, medium-, and high-dose groups versus the model group (*P* < 0.05), with progressive enhancement observed across the dose gradient—most pronounced in the high-dose group, which showed significantly greater viability than both the low- and medium-dose groups (*P* < 0.05). These results demonstrate that Poria cocos triterpenoids confer concentration-dependent protection against hypoxic injury in H9c2 cardiomyoblasts (Table 1).

**Table 1:** CCK-8 assay of cell viability in H9c2 cells of each group.

Groups	Cell viability (%)
Normal control group	100.00 ± 5.21
Model group	56.84 ± 4.67
Low-dose Pachymaran group	67.42 ± 5.03
Medium-dose Pachymaran group	78.96 ± 5.41
High-dose Pachymaran group	88.31 ± 4.88
*F* value	33.880
*P* value	<0.001

### 2.2 Western blot was used to detect the expression of mitochondrial dynamics proteins

**Figure 1:**
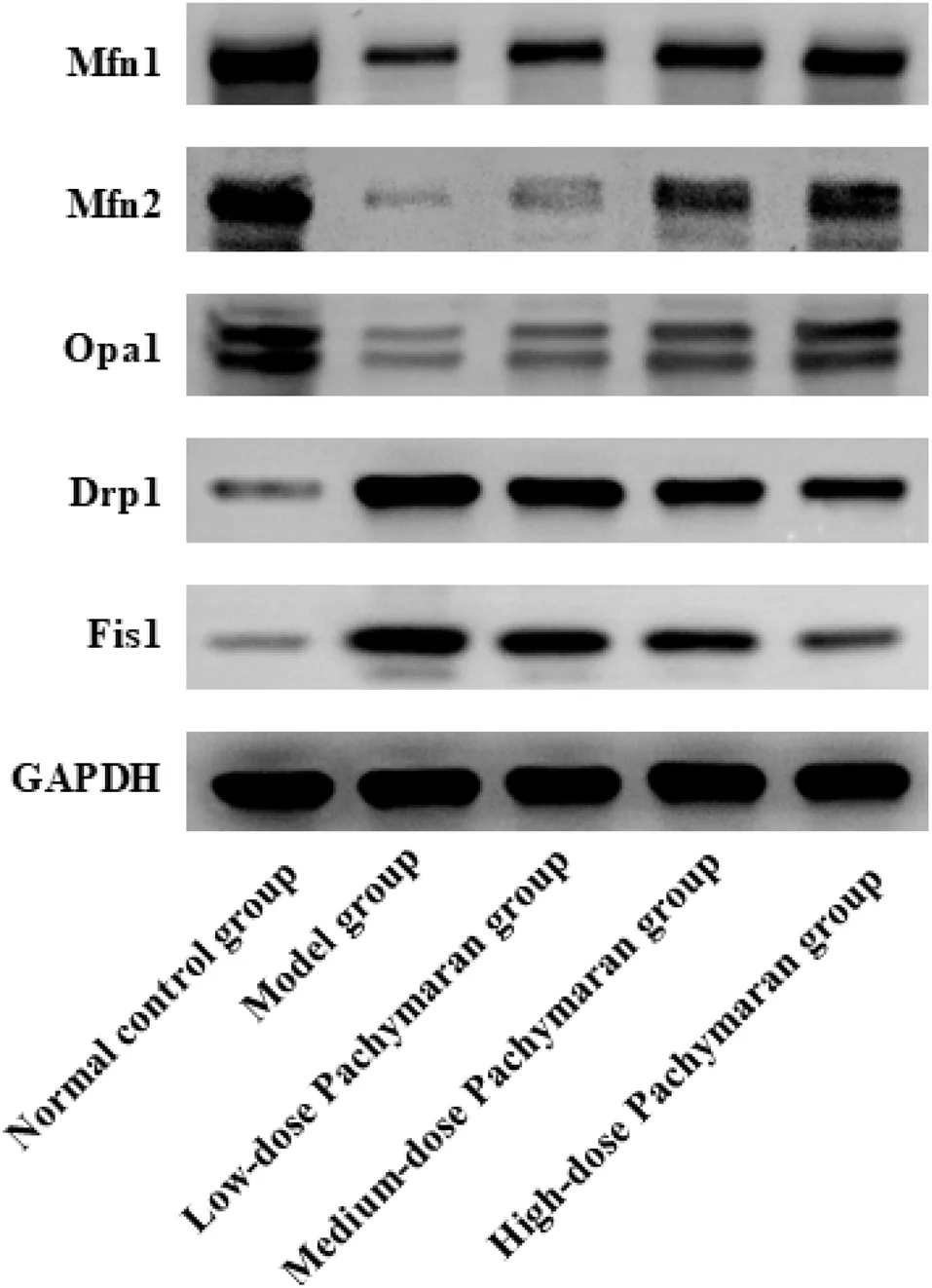
Western blot detection of mitochondrial dynamics protein expression.

Western blot analysis revealed dysregulation of mitochondrial dynamics proteins under hypoxia, which was reversed by Poria cocos triterpenoid treatment. Compared with the normal control group, the model group exhibited significantly decreased expression of mitochondrial fusion proteins Mfn1, Mfn2, and OPA1 (*P* < 0.05), alongside increased expression of fission mediators Drp1 and Fis1 (*P* < 0.05). Triterpenoid treatment dose-dependently restored this imbalance: Mfn1, Mfn2, and OPA1 levels were significantly elevated—and Drp1 and Fis1 levels significantly suppressed—in the low-, medium-, and high-dose groups relative to the model group (*P* < 0.05), with the most robust normalization observed in the high-dose group (Figure 1; Table 2).

**Table 2:** Western blot detection of mitochondrial dynamics protein expression.

Groups	Mfn1	Mfn2	Opa1	Drp1	Fis1
Normal control group	0.96 ± 0.08	1.02 ± 0.09	0.93 ± 0.07	0.55 ± 0.05	0.49 ± 0.04
Model group	0.48 ± 0.05	0.52 ± 0.06	0.45 ± 0.04	1.21 ± 0.11	1.16 ± 0.09
Low-dose Pachymaran group	0.61 ± 0.06	0.66 ± 0.05	0.58 ± 0.05	1.02 ± 0.09	0.96 ± 0.08
Medium-dose Pachymaran group	0.74 ± 0.07	0.81 ± 0.07	0.72 ± 0.06	0.84 ± 0.08	0.78 ± 0.07
High-dose Pachymaran group	0.88 ± 0.06	0.95 ± 0.08	0.86 ± 0.07	0.67 ± 0.06	0.59 ± 0.05
*F* value	27.200	24.806	33.317	32.188	47.189
*P* value	<0.001	<0.001	<0.001	<0.001	<0.001

### 2.3 Comparison of mitochondrial function and levels of metabolic products

The mitochondrial function and the level of metabolic products of each group were compared. The respiratory control ratio (RCR), the ATP synthesis rate and the pyruvate level in the model group was lower than that in the normal control group (*P* < 0.05), and the lactate level was higher than that in the normal control group (*P* < 0.05). The RCR, the ATP synthesis rate and the pyruvate level in the low-dose, medium-dose and high-dose groups of Poria triterpenoids were higher than that in the model group (*P* < 0.05), and the lactate level was lower than that in the model group (*P* < 0.05). Among them, the improvement effect of the high-dose group of Poria triterpenoids was more significant than that of the low-dose and medium-dose groups (*P* < 0.05). Hypoxia can lead to a decline in the mitochondrial respiratory function in the cardiomyocytes and promote the enhancement of the anaerobic glycolysis. Poria triterpenoids can improve the energy generation capacity of the mitochondria and alleviate the accumulation of the metabolic abnormalities (Table 3).

**Table 3:** Comparison of mitochondrial function and metabolic product levels.

Groups	Mitochondrial respiratory control ratio (RCR)	ATP synthesis rate (nmol/min/mg prot)	Lactate (mmol/L)	Pyruvate (μmol/L)
Normal control group	5.12 ± 0.43	42.38 ± 3.56	2.14 ± 0.18	126.43 ± 10.57
Model group	2.36 ± 0.21	21.47 ± 2.14	4.86 ± 0.41	71.28 ± 6.31
Low-dose Pachymaran group	2.91 ± 0.24	27.96 ± 2.42	4.21 ± 0.36	83.56 ± 7.14
Medium-dose Pachymaran group	3.68 ± 0.31	33.84 ± 2.77	3.47 ± 0.29	98.42 ± 8.36
High-dose Pachymaran group	4.42 ± 0.39	39.75 ± 3.18	2.72 ± 0.24	114.79 ± 9.45
*F* value	34.743	26.828	38.236	20.826
*P* value	<0.001	<0.001	<0.001	<0.001

### 2.4 Western blot was used to detect the expression of autophagy-lysosome pathway proteins in each group

**Figure 2:**
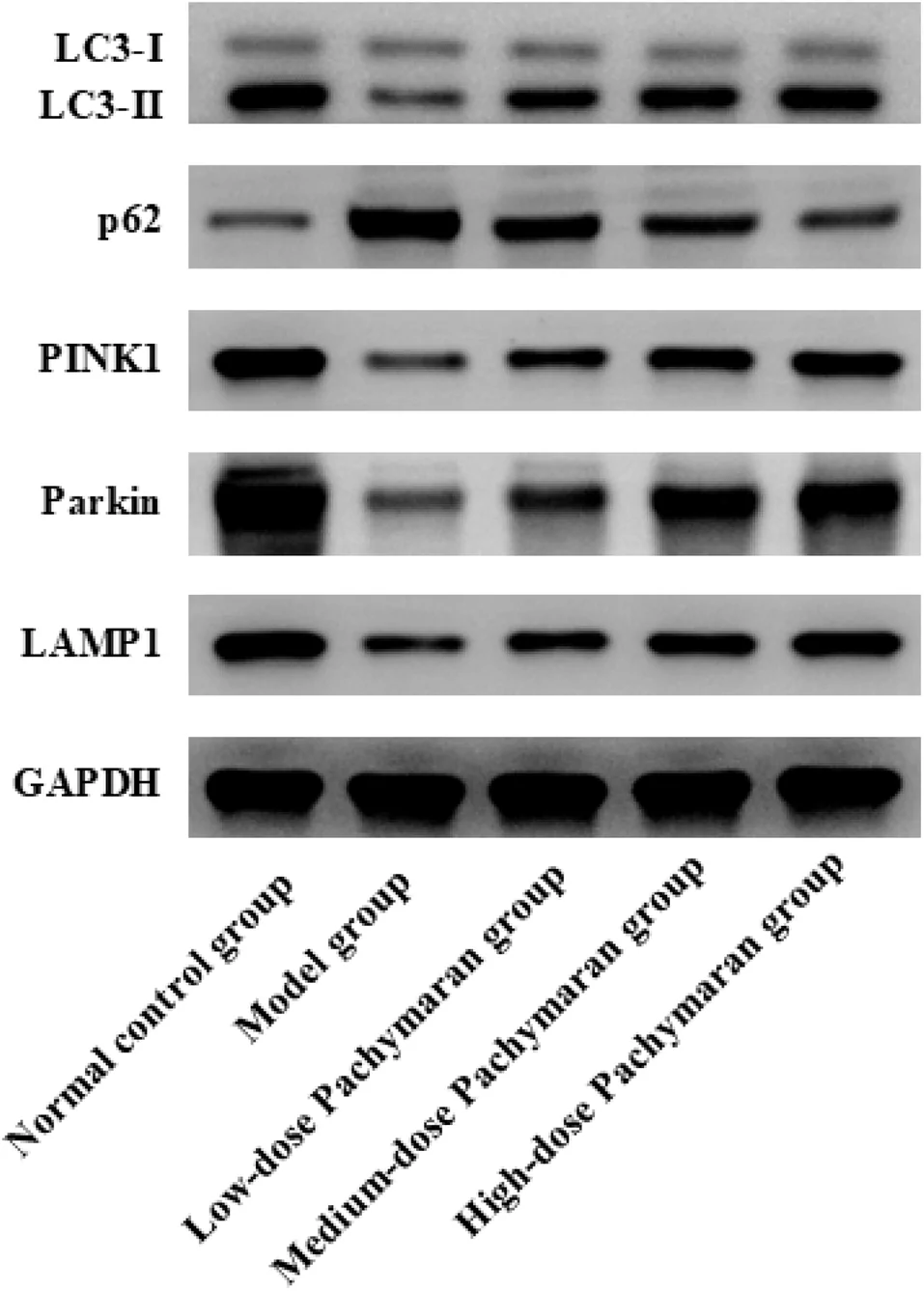
Western blot was used to detect the expression of autophagy-lysosome pathway proteins in each group.

Mitochondrial bioenergetic function and key metabolic intermediates were quantitatively assessed across experimental groups. Relative to the normal control group, the hypoxia model group exhibited significantly impaired mitochondrial respiratory capacity—evidenced by reduced respiratory control ratio (RCR) and ATP synthesis rate—as well as a marked shift toward anaerobic metabolism, reflected by decreased pyruvate levels and elevated lactate accumulation (*P* < 0.05). Poria cocos triterpenoid treatment dose-dependently rescued these deficits: RCR, ATP synthesis rate, and pyruvate levels were significantly increased—and lactate levels significantly decreased—in the low-, medium-, and high-dose groups compared with the model group (*P* < 0.05), with the most pronounced restoration observed in the high-dose group (*P* < 0.05). These findings indicate that hypoxia induces mitochondrial respiratory insufficiency and glycolytic dominance in cardiomyoblasts, and that Poria cocos triterpenoids restore oxidative phosphorylation capacity while suppressing pathological lactate accumulation—thereby ameliorating hypoxia-driven energetic and metabolic dysfunction (Figure 2; Table 4).

**Table 4:** Western blot was used to detect the expression of autophagy-lysosome pathway proteins in each group.

Groups	LC3-Ⅱ/Ⅰ	p62	PINK1	Parkin	LAMP1
Normal control group	1.02 ± 0.09	0.48 ± 0.04	0.94 ± 0.08	0.91 ± 0.07	0.97 ± 0.08
Model group	0.46 ± 0.04	1.19 ± 0.12	0.43 ± 0.04	0.41 ± 0.04	0.52 ± 0.05
Low-dose Pachymaran group	0.61 ± 0.05	0.98 ± 0.08	0.57 ± 0.05	0.55 ± 0.05	0.65 ± 0.06
Medium-dose Pachymaran group	0.79 ± 0.07	0.76 ± 0.06	0.71 ± 0.06	0.69 ± 0.06	0.80 ± 0.07
High-dose Pachymaran group	0.93 ± 0.08	0.59 ± 0.05	0.86 ± 0.07	0.83 ± 0.07	0.92 ± 0.08
*F* value	33.491	43.763	34.161	35.417	22.103
*P* value	<0.001	<0.001	<0.001	<0.001	<0.001

### 2.5 Seahorse analysis of comparisons of OCR, ECAR and fatty acid oxidation efficiency among each group

Extracellular acidification rate (ECAR), oxygen consumption rate (OCR), and fatty acid oxidation (FAO) efficiency were quantified using the Seahorse XFe96 Analyzer. Compared with the normal control group, the hypoxia model group exhibited significantly suppressed mitochondrial respiration (reduced OCR) and impaired FAO capacity, alongside elevated ECAR—indicative of enhanced glycolytic flux (*P* < 0.05). Poria cocos triterpenoid treatment dose-dependently reversed these bioenergetic perturbations: OCR and FAO efficiency were significantly increased—and ECAR significantly decreased—in the low-, medium-, and high-dose groups relative to the model group (*P* < 0.05), with the most substantial restoration observed in the high-dose group (Table 5).

**Table 5:** Comparison of OCR, ECAR and fatty acid oxidation efficiency among groups analyzed by seahorse.

Groups	OCR (pmol/min)	ECAR (mpH/min)	Fatty acid oxidation efficiency (%)
Normal control group	165.42 ± 13.58	24.18 ± 2.15	78.65 ± 6.42
Model group	91.36 ± 8.42	46.28 ± 3.94	42.31 ± 3.68
Low-dose Pachymaran group	109.54 ± 9.37	39.15 ± 3.36	51.73 ± 4.27
Medium-dose Pachymaran group	128.67 ± 10.84	33.64 ± 2.87	62.88 ± 5.14
High-dose Pachymaran group	148.23 ± 12.65	28.96 ± 2.44	71.54 ± 6.08
*F* value	21.094	24.518	23.662
*P* value	<0.001	<0.001	<0.001

### 2.6 Comparison of key metabolic enzyme activities

Activities of key metabolic enzymes were measured spectrophotometrically. Relative to the normal control group, the hypoxia model group exhibited significantly reduced carnitine palmitoyltransferase 1 (CPT1) activity—reflecting impaired mitochondrial fatty acid import—and elevated 6-phosphofructokinase-2 (PFK-2) activity, a critical regulator of glycolytic flux (*P* < 0.05). Poria cocos triterpenoid treatment dose-dependently normalized these enzymatic alterations: CPT1 activity was significantly increased—and PFK-2 activity significantly decreased—in the low-, medium-, and high-dose groups compared with the model group (*P* < 0.05), with maximal correction achieved in the high-dose group. These results demonstrate that hypoxia suppresses fatty acid oxidation while hyperactivating glycolysis at the enzymatic level, and that Poria cocos triterpenoids restore metabolic flexibility in cardiomyoblasts by coordinately enhancing lipid substrate utilization and restraining pathological glycolytic drive (Table 6).

**Table 6:** Comparison of key metabolic enzyme activities.

Groups	CPT1 activity (U/mg prot)	PFK-2 activity (U/mg prot)
Normal control group	18.43 ± 1.56	10.37 ± 0.93
Model group	9.26 ± 0.82	19.82 ± 1.76
Low-dose Pachymaran group	11.45 ± 0.97	17.34 ± 1.51
Medium-dose Pachymaran group	14.17 ± 1.23	14.62 ± 1.28
High-dose Pachymaran group	16.75 ± 1.41	12.48 ± 1.06
*F* value	27.915	23.576
*P* value	<0.001	<0.001

### 2.7 Expression of Drp1/ATG5 and autophagy-related proteins after siRNA transfection

**Figure 3:**
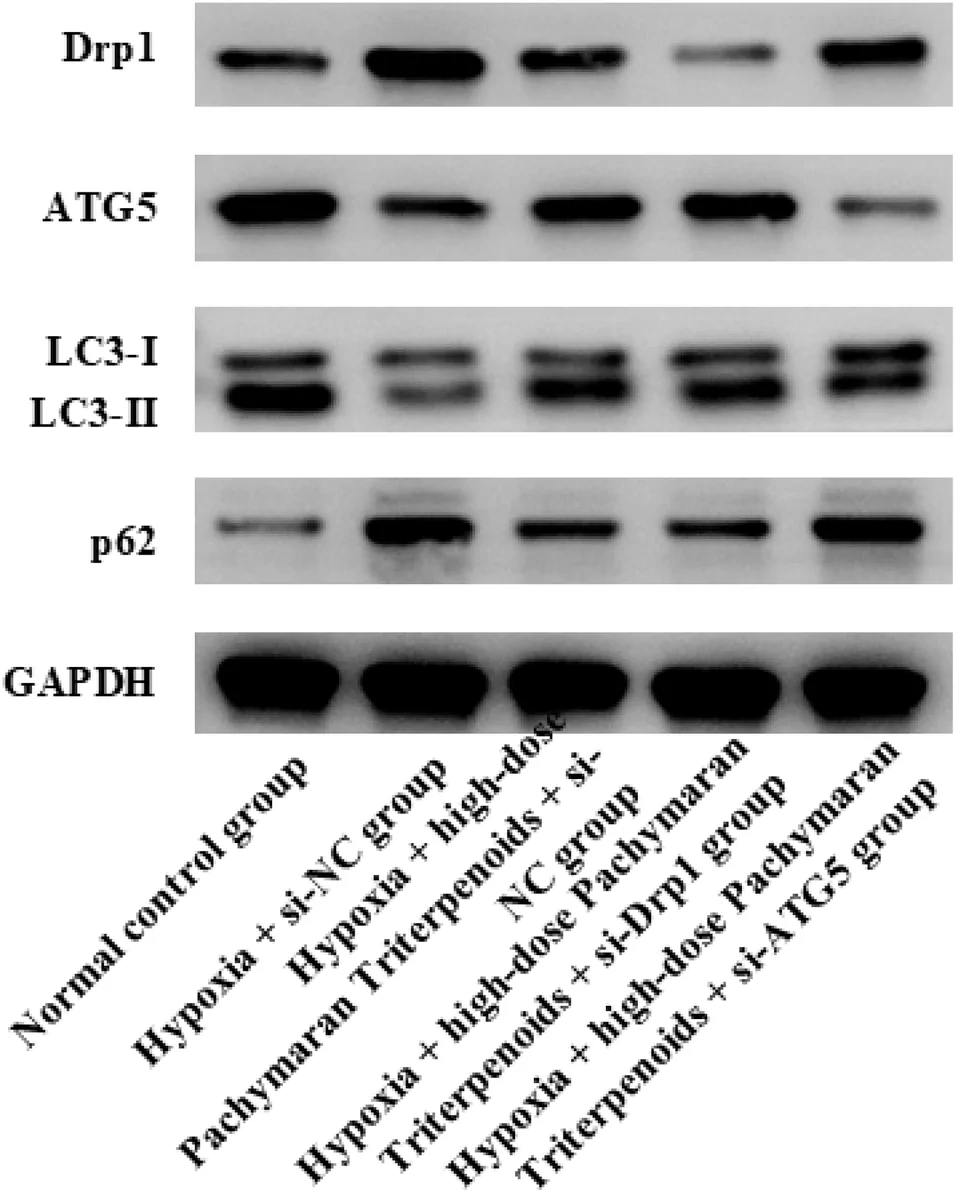
Expression of Drp1/ATG5 and autophagy-related proteins after siRNA transfection.

In the hypoxia + si-NC group, Drp1 expression was significantly upregulated compared with the normal control group (*P* < 0.05), whereas ATG5 and LC3-II/I levels were significantly downregulated (*P* < 0.05), and p62 accumulation was markedly increased (*P* < 0.05), indicating impaired autophagic flux. In the hypoxia + high-dose Pachymaran Triterpenoids + si-NC group, these abnormalities were significantly reversed relative to the hypoxia + si-NC group (*P* < 0.05): Drp1 expression decreased, ATG5 and LC3-II/I levels increased, and p62 accumulation declined. In the hypoxia + high-dose Pachymaran Triterpenoids + si-Drp1 group, Drp1 expression was further suppressed (*P* < 0.05) compared with the hypoxia + high-dose Pachymaran Triterpenoids + si-NC group; however, LC3-II/I and p62 levels showed no significant change, suggesting that Drp1 knockdown alone does not further restore autophagic flux beyond the effect of Pachymaran Triterpenoids. In contrast, in the hypoxia + high-dose Pachymaran Triterpenoids + si-ATG5 group, ATG5 expression was significantly reduced (*P* < 0.05), accompanied by decreased LC3-II/I conversion and elevated p62 accumulation (*P* < 0.05), confirming blockade of autophagosome formation. Collectively, these findings demonstrate that Pachymaran Triterpenoids ameliorate hypoxia-induced mitochondrial fission dysregulation and autophagic flux impairment, and that ATG5 is essential for mediating the compound's restorative effect on the autophagy–lysosome pathway (Figure 3; Table 7).

**Table 7:** Expression of Drp1/ATG5 and autophagy-related proteins after siRNA transfection.

Groups	Drp1	ATG5	LC3-Ⅱ/Ⅰ	p62
Normal control group	0.56 ± 0.05	0.94 ± 0.08	1.01 ± 0.09	0.47 ± 0.04
Hypoxia + si-NC group	1.20 ± 0.10	0.45 ± 0.04	0.48 ± 0.04	1.17 ± 0.11
Hypoxia + high-dose Pachymaran Triterpenoids + si-NC group	0.68 ± 0.06	0.85 ± 0.07	0.92 ± 0.08	0.61 ± 0.05
Hypoxia + high-dose Pachymaran Triterpenoids + si-Drp1 group	0.34 ± 0.03	0.84 ± 0.07	0.95 ± 0.08	0.58 ± 0.05
Hypoxia + high-dose Pachymaran Triterpenoids + si-ATG5 group	0.71 ± 0.06	0.31 ± 0.03	0.49 ± 0.05	1.06 ± 0.09
*F* value	72.757	62.543	41.250	55.338
*P* value	<0.001	<0.001	<0.001	<0.001

### 2.8 Comparison of cell viability and seahorse metabolic indicators

In the hypoxia + si-NC group, cell viability and oxygen consumption rate (OCR) were significantly reduced compared with the normal control group (*P* < 0.05), whereas the extracellular acidification rate (ECAR) was significantly elevated (*P* < 0.05), indicating a metabolic shift from oxidative phosphorylation to glycolysis. In the hypoxia + high-dose Pachymaran Triterpenoids + si-NC group, cell viability and OCR were significantly restored relative to the hypoxia + si-NC group (*P* < 0.05), and ECAR was correspondingly decreased (*P* < 0.05), suggesting partial reversal of hypoxia-induced metabolic reprogramming. Notably, in the hypoxia + high-dose Pachymaran Triterpenoids + si-Drp1 group, cell viability and OCR were further increased—and ECAR further decreased—compared with the hypoxia + high-dose Pachymaran Triterpenoids + si-NC group (*P* < 0.05), implying that Drp1 knockdown synergizes with Pachymaran to enhance mitochondrial respiration and suppress glycolytic dependency. Conversely, in the hypoxia + high-dose Pachymaran Triterpenoids + si-ATG5 group, cell viability and OCR were significantly attenuated, and ECAR was elevated relative to the hypoxia + high-dose Pachymaran Triterpenoids + si-NC group (*P* < 0.05), demonstrating that ATG5 deficiency abolishes the metabolic protective effects of Pachymaran. Collectively, these data indicate that Pachymaran Triterpenoids ameliorate hypoxia-induced bioenergetic failure in cardiomyocytes through a mechanism dependent on ATG5-mediated autophagy and modulated by Drp1-regulated mitochondrial dynamics (Table 8).

**Table 8:** Comparison of cell viability and seahorse metabolic indicators.

Groups	Cell viability (%)	OCR (pmol/min)	ECAR (mpH/min)
Normal control group	100.00 ± 5.18	166.28 ± 13.47	24.05 ± 2.11
Hypoxia + si-NC group	57.26 ± 4.82	92.18 ± 8.54	45.86 ± 3.87
Hypoxia + high-dose Pachymaran Triterpenoids + si-NC group	88.14 ± 5.06	149.36 ± 12.44	29.18 ± 2.53
Hypoxia + high-dose Pachymaran Triterpenoids + si-Drp1 group	92.37 ± 5.24	156.72 ± 12.85	26.84 ± 2.32
Hypoxia + high-dose Pachymaran Triterpenoids + si-ATG5 group	68.53 ± 4.96	112.64 ± 9.83	39.74 ± 3.35
*F* value	36.993	22.297	30.343
*P* value	<0.001	<0.001	<0.001

### 2.9 Comparison of ATP synthesis rate and key metabolic enzyme activities

In the hypoxia + si-NC group, the ATP synthesis rate and carnitine palmitoyltransferase 1 (CPT1) activity were significantly reduced compared with the normal control group (*P* < 0.05), whereas 6-phosphofructo-2-kinase (PFK-2) activity was significantly elevated (*P* < 0.05), reflecting suppressed mitochondrial fatty acid oxidation and enhanced glycolytic flux. In the hypoxia + high-dose Poria triterpenoids + si-NC group, both ATP synthesis rate and CPT1 activity were significantly restored relative to the hypoxia + si-NC group (*P* < 0.05), and PFK-2 activity was correspondingly suppressed (*P* < 0.05), indicating that Poria triterpenoids partially reverse hypoxia-induced metabolic remodeling. Notably, in the hypoxia + high-dose Poria triterpenoids + si-Drp1 group, ATP synthesis rate and CPT1 activity were further increased—and PFK-2 activity further decreased—compared with the hypoxia + high-dose Poria triterpenoids + si-NC group (*P* < 0.05), suggesting that Drp1 knockdown potentiates the compound's ability to promote mitochondrial substrate utilization and inhibit glycolysis. In contrast, in the hypoxia + high-dose Poria triterpenoids + si-ATG5 group, ATP synthesis rate and CPT1 activity were significantly attenuated, and PFK-2 activity was elevated relative to the hypoxia + high-dose Poria triterpenoids + si-NC group (*P* < 0.05), confirming that ATG5 is required for Poria triterpenoids to sustain mitochondrial energy production and restrain excessive glycolysis (Table 9).

**Table 9:** Comparison of ATP synthesis rate and Key metabolic enzyme activities.

Groups	ATP synthesis rate (nmol/min/mg prot)	CPT1 activity (U/mg prot)	PFK-2 activity (U/mg prot)
Normal control group	42.16 ± 3.48	18.36 ± 1.55	10.28 ± 0.91
Hypoxia + si-NC group	21.74 ± 2.09	9.41 ± 0.84	19.56 ± 1.71
Hypoxia + high-dose Pachymaran Triterpenoids + si-NC group	39.21 ± 3.17	16.68 ± 1.39	12.63 ± 1.08
Hypoxia + high-dose Pachymaran Triterpenoids + si-Drp1 group	41.63 ± 3.26	17.42 ± 1.46	11.84 ± 1.02
Hypoxia + high-dose Pachymaran Triterpenoids + si-ATG5 group	28.36 ± 2.41	12.36 ± 1.07	16.92 ± 1.43
*F* value	28.991	26.183	27.886
*P* value	<0.001	<0.001	<0.001

### 2.10 Comparison of fatty acid oxidation efficiency and lactate levels among groups

Fatty acid oxidation (FAO) efficiency and lactate concentration were assessed across experimental groups. In the hypoxia + si-NC group, FAO efficiency was significantly decreased compared with the normal control group (*P* < 0.05), while lactate accumulation was significantly elevated (*P* < 0.05), consistent with impaired mitochondrial lipid metabolism and compensatory glycolytic activation. In the hypoxia + high-dose Poria tetracyclic triterpenoids + si-NC group, FAO efficiency was significantly restored—and lactate levels significantly reduced—relative to the hypoxia + si-NC group (*P* < 0.05), indicating that Poria tetracyclic triterpenoids ameliorate hypoxia-induced metabolic dysfunction. Further, in the hypoxia + high-dose Poria tetracyclic triterpenoids + si-Drp1 group, FAO efficiency was further enhanced and lactate accumulation further suppressed compared with the hypoxia + high-dose Poria tetracyclic triterpenoids + si-NC group (*P* < 0.05), demonstrating that Drp1 knockdown synergistically augments the compound's capacity to promote mitochondrial fatty acid utilization and limit anaerobic glycolysis. Conversely, in the hypoxia + high-dose Poria tetracyclic triterpenoids + si-ATG5 group, FAO efficiency was significantly diminished and lactate levels significantly increased relative to the hypoxia + high-dose Poria tetracyclic triterpenoids + si-NC group (*P* < 0.05), confirming that ATG5 is indispensable for the metabolic restorative effects of Poria tetracyclic triterpenoids (Table 10).

**Table 10:** Comparison of fatty acid oxidation efficiency and lactic acid levels among groups.

Groups	Fatty acid oxidation efficiency (%)	Lactic acid (mmol/L)
Normal control group	79.24 ± 6.38	2.16 ± 0.19
Hypoxia + si-NC group	43.02 ± 3.76	4.79 ± 0.41
Hypoxia + high-dose Pachymaran Triterpenoids + si-NC group	72.16 ± 6.07	2.75 ± 0.24
Hypoxia + high-dose Pachymaran Triterpenoids + si-Drp1 group	75.48 ± 6.15	2.48 ± 0.21
Hypoxia + high-dose Pachymaran Triterpenoids + si-ATG5 group	54.37 ± 4.62	3.96 ± 0.34
*F* value	23.861	43.632
*P* value	<0.001	<0.001

### 2.11 Comparison of energy metabolism remodeling-related indicators among each group

Cell viability, ATP synthesis rate, and lactate concentration were quantified across all experimental groups. In the hypoxia + si-NC group, both cell viability and ATP synthesis rate were significantly reduced compared with the normal control group (*P* < 0.05), whereas lactate concentration was significantly elevated (*P* < 0.05), indicating bioenergetic failure and compensatory glycolytic upregulation under hypoxia. In the hypoxia + high-dose Pachymaran Triterpenoids + si-NC group, cell viability and ATP synthesis rate were significantly restored—and lactate concentration significantly decreased—relative to the hypoxia + si-NC group (*P* < 0.05), demonstrating that Pachymaran Triterpenoids mitigate hypoxia-induced metabolic impairment. Notably, the hypoxia + high-dose Pachymaran Triterpenoids + si-Drp1 group exhibited the greatest improvement: cell viability and ATP synthesis rate were further increased, and lactate concentration further reduced, compared with the hypoxia + high-dose Pachymaran Triterpenoids + si-NC group (*P* < 0.05). In contrast, in the hypoxia + high-dose Pachymaran Triterpenoids + si-ATG5 group, cell viability and ATP synthesis rate were significantly attenuated, and lactate concentration significantly increased relative to the hypoxia + high-dose Pachymaran Triterpenoids + si-NC group (*P* < 0.05), confirming that ATG5 is essential for the compound's protective effects on cellular energetics (Table 11).

**Table 11:** Comparison of energy metabolism remodeling-related indicators among groups.

Groups	Cell viability (%)	Adenosine triphosphate (ATP) synthesis rate (nmol/min/mg prot)	Lactate (mmol/L)
Normal control group	100.00 ± 5.18	42.16 ± 3.48	2.16 ± 0.19
Hypoxia + si-NC group	57.26 ± 4.82	21.74 ± 2.09	4.79 ± 0.40
Hypoxia + high-dose Pachymaran Triterpenoids + si-NC group	88.14 ± 5.06	39.21 ± 3.17	2.75 ± 0.24
Hypoxia + high-dose Pachymaran Triterpenoids + si-Drp1 group	92.37 ± 5.24	41.63 ± 3.26	2.48 ± 0.21
Hypoxia + high-dose Pachymaran Triterpenoids + si-ATG5 group	68.53 ± 4.96	28.36 ± 2.41	3.96 ± 0.34
*F* value	36.993	28.991	44.487
*P* value	<0.001	<0.001	<0.001

### 2.12 Mitochondrial morphology under transmission electron microscopy

**Figure 4:**
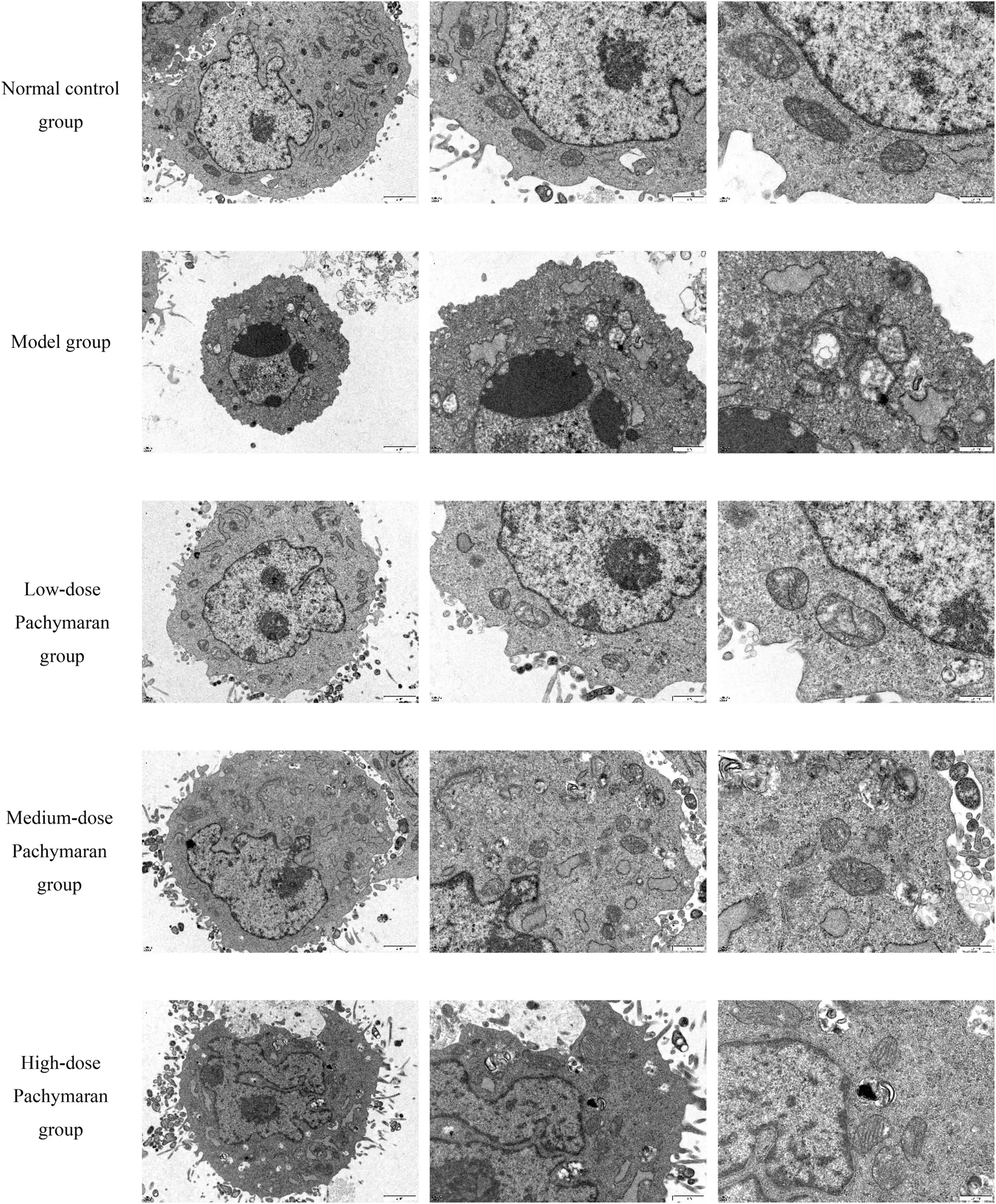
Expression of Drp1/ATG5 and autophagy-related proteins after siRNA transfection.

Compared to the normal control group, H9c2 cardiac myocytes in the hypoxia model group exhibited significant mitochondrial morphological abnormalities: overall mitochondrial swelling, disrupted and dissolved cristae, markedly increased vacuolization, significantly shortened average mitochondrial length, a notable increase in fragmented mitochondria, and accumulation of large numbers of damaged mitochondria that failed to be cleared. After intervention with different doses of poria triterpenoids, these abnormalities showed dose-dependent improvement: mitochondrial swelling was significantly reduced in the medium- and high-dose groups, cristae structure became more orderly, and the proportion of fragmented mitochondria gradually decreased; the high-dose group approached the mitochondrial morphology characteristics of the normal control group. When Drp1 was knocked down in combination with high-dose drug treatment, mitochondrial fusion was further enhanced, and the proportion of long tubular mitochondria increased significantly. In contrast, after ATG5 knockdown, the morphological protective effect of poria triterpenoids was almost completely lost, and numerous swollen, vacuolated damaged mitochondria could not be cleared via autophagy (Figure 4; Table 12).

**Table 12:** Mitochondrial morphology under transmission electron microscopy.

Groups	Average mitochondrial length (μm)	Percentage of fragmented mitochondria (%)	Percentage of vacuolated mitochondria (%)	Percentage of mitochondria in complete ridge structure (%)
Normal control group	3.72 ± 0.41	8.25 ± 1.36	6.18 ± 1.07	91.36 ± 3.25
Model group	1.28 ± 0.27	76.43 ± 5.28	68.74 ± 4.62	22.57 ± 3.18
Low-dose Pachymaran group	1.85 ± 0.32	52.67 ± 4.35	45.29 ± 3.71	47.62 ± 4.23
Medium-dose Pachymaran group	2.64 ± 0.38	31.52 ± 3.64	24.85 ± 2.96	72.48 ± 3.87
High-dose Pachymaran group	3.37 ± 0.45	14.76 ± 2.19	11.32 ± 1.85	85.73 ± 3.54
*F* value	127.634	215.742	189.367	242.581
*P* value	<0.001	<0.001	<0.001	<0.001

## 3 Discussion

Cardiomyocytes exhibit exceptionally high energy demands, and mitochondria serve not only as the primary site of ATP production but also as central regulators of redox homeostasis, calcium signaling, and cell fate decisions. Substantial evidence demonstrates that mitochondrial dysfunction under hypoxic or ischemic conditions directly impairs oxidative phosphorylation, amplifies reactive oxygen species (ROS) generation, and shifts substrate utilization—thereby accelerating myocardial injury (19, 20). Increasingly, dysregulated mitochondrial dynamics and defective mitophagy are recognized as mechanistically upstream drivers of cardiovascular disease progression, establishing mitochondrial quality control (MQC) as a pivotal therapeutic target in hypoxic myocardial injury (21).

In this study, hypoxia exposure significantly reduced H9c2 cardiomyoblast viability and impaired multiple bioenergetic parameters: respiratory control ratio (RCR), ATP synthesis rate, oxygen consumption rate (OCR), fatty acid oxidation (FAO) efficiency, and carnitine palmitoyltransferase 1 (CPT1) activity—all decreased (*P* < 0.05); conversely, lactate concentration, extracellular acidification rate (ECAR), and 6-phosphofructo-2-kinase (PFK-2) activity were significantly elevated (*P* < 0.05). These coordinated alterations reflect a hallmark metabolic reprogramming—suppression of mitochondrial oxidative metabolism coupled with compensatory glycolytic activation. Dose-dependent treatment with *Poria cocos* triterpenoids progressively reversed these deficits, with the high-dose group achieving the most complete restoration of bioenergetic function, thereby demonstrating potent and graded metabolic protection against hypoxic stress. This effect is consistent with the compound's capacity to ameliorate mitochondrial energetic insufficiency and restore physiological substrate preference. Notably, pachymic acid A—the principal bioactive triterpenoid in *Poria cocos*—has been shown to attenuate hypoxia-induced injury in primary cardiomyocytes and enhance mitochondrial respiratory capacity. Moreover, it promotes autophagic flux and improves post-infarction cardiac repair via AMPK/mTOR pathway activation. Our data extend these findings by confirming that Poria triterpenoids confer comprehensive protection: preserving cellular viability, restoring oxidative phosphorylation efficiency, suppressing pathological lactate accumulation, and rescuing FAO capacity.

Abnormal mitochondrial dynamics constitute a core pathogenic mechanism in this model. Under physiological conditions, mitochondrial network plasticity is maintained by balanced fission—mediated by Drp1 and Fis1—and fusion—mediated by Mfn1, Mfn2, and Opa1—to meet dynamic cellular energy and stress demands. Hypoxia disrupts this equilibrium, inducing Drp1/Fis1-dependent hyperfission while suppressing Mfn1/Mfn2/Opa1-mediated fusion. This imbalance drives mitochondrial fragmentation, collapse of mitochondrial membrane potential (Δ*Ψ*m), and consequent respiratory failure. Prior studies demonstrate that Opa1 overexpression reduces hypoxia-induced cardiomyocyte death; conversely, downregulation of Mfn2 and Opa1 correlates strongly with pathological remodeling, mitochondrial fragmentation, and systolic dysfunction. A recent comprehensive review further establishes Drp1-driven hyperfission as a conserved feature of mitochondrial dysfunction across diverse cardiac pathologies (22, 23). Consistent with these reports, our hypoxic model exhibited significant downregulation of Mfn1, Mfn2, and Opa1, alongside marked upregulation of Drp1 and Fis1. Poria triterpenoid treatment fully reversed this dysregulation—increasing expression of fusion proteins and decreasing fission protein levels—thereby restoring mitochondrial structural integrity and functional competence, as confirmed by recovery of RCR, ATP synthesis rate, and OCR. This indicates that the improvement of mitochondrial dynamics is related to the enhancement of the cell's energy supply function.

Mitochondrial quality control critically depends on efficient mitophagy, whose functional completion requires both autophagosome formation and lysosomal degradation. Critically, autophagic flux—not isolated marker expression—determines biological outcome. Although autophagosome biogenesis may increase during ischemia–reperfusion, lysosomal degradation often remains impaired; for example, reduced lysosome-associated membrane protein 1 (LAMP1) expression exacerbates cardiomyocyte injury. Moreover, p62 accumulation can stabilize HIF-1*α* and Nrf2 under hypoxia, underscoring the necessity of interpreting autophagy substrates within the context of overall flux status. In our model, hypoxia induced concurrent reductions in LC3-II/I ratio, PINK1, Parkin, and LAMP1, accompanied by p62 accumulation—indicating dual defects: insufficient initiation of mitophagy and compromised lysosomal degradative capacity. Consequently, damaged mitochondria and misfolded proteins accumulate. Poria triterpenoid treatment completely rescued this phenotype: LC3-II/I ratio, PINK1, Parkin, and LAMP1 levels increased, while p62 decreased—demonstrating restored PINK1/Parkin-mediated mitophagic initiation and lysosomal functionality. Given that PINK1/Parkin signaling maintains cardiomyocyte homeostasis and mitigates ischemic damage—and that pachymic acid A enhances autophagic flux in myocardial tissue—these findings position enhanced mitophagic flux as an essential and non-redundant protective mechanism.

Mechanistic validation revealed that Drp1 knockdown synergized with Poria triterpenoids: cell viability, OCR, ATP synthesis rate, FAO efficiency, and CPT1 activity improved significantly beyond the level achieved by triterpenoids alone, while ECAR, PFK-2 activity, and lactate concentration declined further. This indicates that Drp1-mediated hyperfission is a primary driver of bioenergetic collapse in hypoxia, and its targeted attenuation potentiates the compound's efficacy. Prior studies corroborate that Drp1 suppression rescues mitochondrial respiration in H9c2 cells, and numerous natural products exert cardioprotection partly through restraining Drp1-dependent pathological fission (24, 25). Crucially, however, Drp1 is not exclusively pathological: it enables essential physiological processes—including mitochondrial turnover, distribution, and mitophagy initiation. Complete or chronic Drp1 inhibition may therefore impair cardiac homeostasis, highlighting the therapeutic imperative for context-specific, modulated regulation rather than global ablation (26, 27).

ATG5 silencing abolished the metabolic benefits of Poria triterpenoids: LC3-II/I ratio decreased, p62 accumulated, and all previously rescued bioenergetic parameters—viability, OCR, ATP synthesis rate, CPT1 activity, and FAO efficiency—reverted toward hypoxic baseline levels, while ECAR, PFK-2 activity, and lactate concentration rose again. This confirms that ATG5-dependent autophagosome formation is indispensable for the compound's protective action. Literature consistently shows that ATG5 deficiency severely compromises cardiomyocyte survival and autophagic flux during ischemia–reperfusion and hypoxia/reoxygenation (28, 29). Similarly, pharmacological cardioprotection is frequently attenuated when ATG5 is inhibited (9). Our data thus identify ATG5-mediated autophagy as a non-redundant, rate-limiting node through which Poria triterpenoids restore mitochondrial quality and correct metabolic dysfunction. This indicates that the improvement of the cell state is not solely dependent on the reduction of abnormal mitochondrial division. This process is also related to whether the damaged mitochondria can smoothly enter the autophagy-lysosome degradation process (30, 31).

The experimental design of this study has certain limitations. This study relies solely on H9c2 cells exposed to CoCl₂-induced hypoxic conditions, which has certain drawbacks. That is, although this model is widely used, it cannot fully simulate the physiological state of hypoxia. This experiment has limitations in demonstrating autophagic flux. Although the changes in the LC3-II/I ratio, p62, PINK1, Parkin and LAMP1 expressions provide indirect evidence for autophagic flux, they do not conclusively prove the existence of autophagic flux. The experiments in this study were all conducted only on the H9c2 cell line (rat cardiomyocytes), which significantly limits its clinical translational value. The study lacks validation in primary cells and in *in vivo* models (such as myocardial ischemia). The current research conclusions mainly concern the cellular mechanisms rather than potential clinical therapeutic applications. In this study, cobalt chloride (CoCl₂) was used as the hypoxia simulator. This is a common solution that cannot fully represent the physiological state of hypoxia and may also cause other effects unrelated to hypoxia. Therefore, the application of this model has certain limitations. Further research will refine the research system by introducing conditions of oxygen deficiency in gases. This study demonstrated the changes in Drp1 and ATG5 levels, but did not analyze the underlying signaling pathways (such as AMPK, mTOR, HIF-1*α*). Due to the length constraints of this study, the mechanism by which the triterpenoid compounds trigger these observed effects was not explained. Therefore, this study is at the descriptive level and will be further refined in future studies to conduct mechanism research.

This study provides integrated experimental evidence that Poria cocos triterpenoids protect hypoxic cardiomyocytes by coordinately targeting two interdependent pillars of mitochondrial quality control: normalizing mitochondrial dynamics via Drp1 downregulation to prevent pathological fragmentation, and sustaining ATG5-dependent autophagic flux to ensure timely clearance of damaged mitochondria. This dual-action mechanism restores oxidative phosphorylation capacity, suppresses maladaptive glycolysis, and preserves cellular viability. While prior studies identified individual bioactivities of Poria constituents—including polysaccharides, sterols, and triterpenoids—our work advances mechanistic understanding by linking pachymic acid A–driven modulation of MQC to systemic metabolic rescue. Unlike reports focusing on isolated inflammatory or apoptotic endpoints, we demonstrate that the compound's efficacy arises from hierarchical correction of mitochondrial structure, turnover, and bioenergetic output—thereby establishing mitochondrial quality control as the unifying mechanistic framework underlying its cardioprotective effects in hypoxia.

## Data Availability

The original contributions presented in the study are included in the article/Supplementary Material, further inquiries can be directed to the corresponding author.
